# Epitranscriptomic regulation of HIF-1: bidirectional regulatory pathways

**DOI:** 10.1186/s10020-025-01149-x

**Published:** 2025-03-18

**Authors:** Daniel Benak, Petra Alanova, Kristyna Holzerova, Miloslava Chalupova, Barbora Opletalova, Frantisek Kolar, Gabriela Pavlinkova, Marketa Hlavackova

**Affiliations:** 1 Laboratory of Developmental Cardiology, Institute of Physiology of the Czech Academy of Sciences, Prague, Czech Republic; 2 Department of Physiology, Faculty of Science, Charles University, Prague, Czech Republic; 3 Laboratory of Molecular Pathogenetics, Institute of Biotechnology, Czech Academy of Sciences, Vestec, Czech Republic

**Keywords:** HIF-1, Hypoxia-inducible factor-1, Epitranscriptomics, m^6^A, Cancer, Heart

## Abstract

**Background:**

Epitranscriptomics, the study of RNA modifications such as N^6^-methyladenosine (m^6^A), provides a novel layer of gene expression regulation with implications for numerous biological processes, including cellular adaptation to hypoxia. Hypoxia-inducible factor-1 (HIF-1), a master regulator of the cellular response to low oxygen, plays a critical role in adaptive and pathological processes, including cancer, ischemic heart disease, and metabolic disorders. Recent discoveries accent the dynamic interplay between m^6^A modifications and HIF-1 signaling, revealing a complex bidirectional regulatory network. While the roles of other RNA modifications in HIF-1 regulation remain largely unexplored, emerging evidence suggests their potential significance.

**Main body:**

This review examines the reciprocal regulation between HIF-1 and epitranscriptomic machinery, including m^6^A writers, readers, and erasers. HIF-1 modulates the expression of key m^6^A components, while its own mRNA is regulated by m^6^A modifications, positioning HIF-1 as both a regulator and a target in this system. This interaction enhances our understanding of cellular hypoxic responses and opens avenues for clinical applications in treating conditions like cancer and ischemic heart disease. Promising progress has been made in developing selective inhibitors targeting the m^6^A-HIF-1 regulatory axis. However, challenges such as off-target effects and the complexity of RNA modification dynamics remain significant barriers to clinical translation.

**Conclusion:**

The intricate interplay between m^6^A and HIF-1 highlights the critical role of epitranscriptomics in hypoxia-driven processes. Further research into these regulatory networks could drive therapeutic innovation in cancer, ischemic heart disease, and other hypoxia-related conditions. Overcoming challenges in specificity and off-target effects will be essential for realizing the potential of these emerging therapies.

## Background

Hypoxia-inducible factor (HIF) has been recognized as a key regulator of the cellular response to low oxygen levels, essential for maintaining oxygen homeostasis (Wang et al. 1995). Hypoxia can occur in both physiological and pathological conditions, positioning HIF at the core of processes such as development (Bohuslavova et al. 2019), metabolism (Kierans and Taylor 2021), angiogenesis (Monaci et al. 2024), and diseases like cancer (Pezzuto and Carico 2018) and ischemic heart disease (Semenza 2014).

HIF is a heterodimer composed of an oxygen-sensitive α-subunit and a constitutively expressed β-subunit (Wang and Semenza 1995). Three paralogues of the HIF-α subunit (HIF-1α, HIF-2α, and HIF-3α), and three paralogues of HIF-β have been identified in mammals (Zagórska and Dulak 2004). Among these, HIF-1α, expressed ubiquitously, plays a central role in regulating the hypoxic response. However, HIF-2α expressed in specific tissues also contributes significantly to this regulation (Wiesener et al. 2003). The function of HIF-3α is less understood, and it has traditionally been considered an inhibitor of HIF-1α activity (Makino et al. 2001). However, its role appears to be isoform-dependent, as some splicing variants have been shown to induce the expression of certain genes, including erythropoietin (EPO) (Tolonen et al. 2020). While HIF activity has traditionally been linked to protein-level regulation and oxygen-dependent mechanisms, recent studies have highlighted the crucial involvement of RNA modifications in its regulation (Nan et al. 2023).

Epitranscriptomics, also known as RNA epigenetics, is the study of chemical modifications on RNA molecules that regulate their function without altering the underlying RNA sequence. These modifications provide an additional layer of control over gene expression, much like epigenetic changes on DNA or histones (Peixoto et al. 2020). By dynamically regulating RNA fate, epitranscriptomic modifications enable cells to fine-tune gene expression in response to environmental cues and cellular signals. These mechanisms are especially critical under conditions of stress or altered metabolism, such as hypoxia, which directly affects HIF-1 signaling pathway (Hlavackova et al. 2018). First discovered in the 1960s, these modifications were initially thought to be adaptive markers that adjust the structure of mature RNA (Davis and Allen 1957; Cohn 1960). Today, over 170 different RNA modifications have been identified (Cappannini et al. 2024). Among these N^6^-methyladenosine (m^6^A) stands out as the most abundant one in mRNA (Desrosiers et al. 1974). m^6^A is implicated in nearly every stage of RNA metabolism, including mRNA translation, degradation, splicing, export, and folding (Longenecker et al. 2020). By modulating the activity of target transcripts, m^6^A subsequently influences various cellular processes and physiological functions. Importantly, it is a reversible and dynamic modification that responds to environmental conditions (Batista et al. 2014). Dysregulation of m^6^A signaling has been linked to many severe human diseases, including neurodegenerative, metabolic, and cardiovascular disorders (Meier et al. 2016).

Recent studies have uncovered a complex, bidirectional relationship between m^6^A modifications and HIF-1 signaling, in which m^6^A regulates *HIF1A* expression (Liang et al. 2022), while HIF-1, in turn, influences the m^6^A machinery (Lu et al. 2024). This review aims to explore the intricate crosstalk between m^6^A and HIF-1, focusing on their reciprocal regulation and its biological and clinical implications. By integrating these findings, we aim to shed light on the growing importance of epitranscriptomics in hypoxic signaling and its therapeutic potential.

## HIF-1

HIF-1 is a heterodimeric transcription factor composed of an oxygen-sensitive HIF-1α subunit and a constitutively expressed HIF-1β subunit, also known as the aryl hydrocarbon receptor nuclear translocator (ARNT) (Wang and Semenza 1995). The activity of HIF-1 is tightly controlled by oxygen-dependent post-translational modifications that regulate the stability and activity of the HIF-1α subunit.

Under normoxic conditions, HIF-1α is rapidly degraded through the ubiquitin–proteasome pathway. This degradation is initiated by proline hydroxylation, which is catalyzed by the prolyl hydroxylase domain (PHD) family of enzymes (PHD1, PHD2, and PHD3) (Epstein et al. 2001). Proline hydroxylation promotes the binding of HIF-1α to the von Hippel-Lindau (pVHL) protein, an E3 ubiquitin ligase that targets HIF-1α for proteasomal degradation. Simultaneously, asparagine hydroxylation by the enzyme factor inhibiting HIF-1 (FIH-1) prevents the recruitment of transcriptional co-activators, further suppressing HIF-1 activity under normal oxygen conditions (Koivunen et al. 2004).

**Fig. 1:**
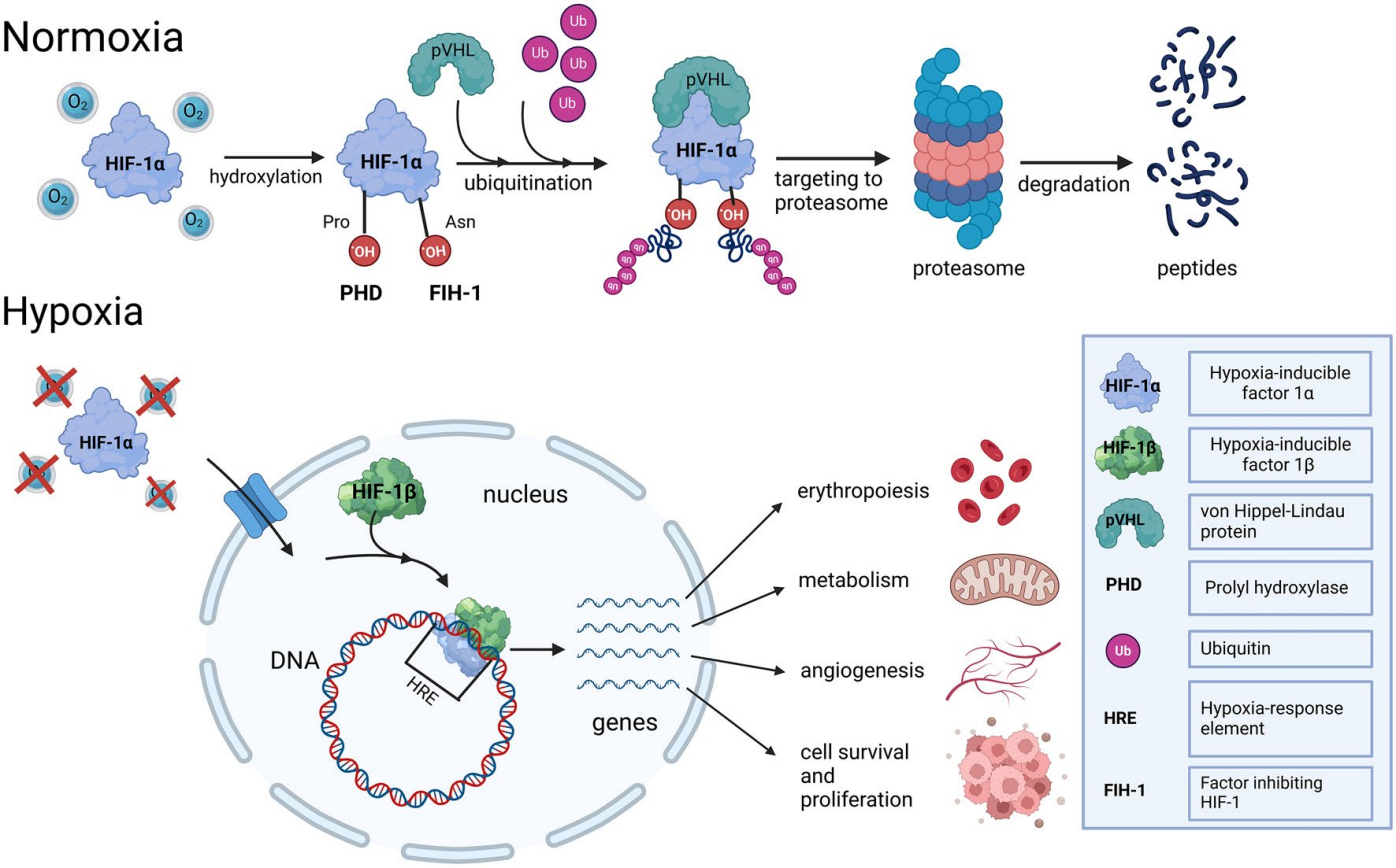
Overview of HIF-1 regulation. Under normoxia, PHDs hydroxylate HIF-1α, triggering pVHL-mediated degradation, while FIH-1 inhibits its transcriptional activity. In hypoxia, HIF-1α is stabilized, translocates into the nucleus, dimerizes with HIF-1β, and binds to HREs to activate genes involved in erythropoiesis, metabolism, angiogenesis, and cell survival

During hypoxia (Fig. 1), reduced oxygen availability inhibits the enzymatic activity of PHDs and FIH. This allows HIF-1α to escape degradation, accumulate in the cytoplasm, and translocate to the nucleus, where it dimerizes with HIF-1β. The HIF-1α/HIF-1β heterodimer binds to hypoxia-response elements (HREs) within the promoters and enhancers of target genes, initiating their transcription (Semenza 2001). The activation of HIF-1 occurs in a stepwise manner. Initially, the inhibition of PHD activity under mild hypoxia allows for the stabilization of HIF-1α. Under more severe hypoxic conditions, FIH-1 activity is also suppressed, enhancing the transactivation potential of HIF-1. This coordinated regulation enables HIF-1 to achieve maximal activity, driving the transcription of a diverse array of genes. These target genes, numbering over 1000, mediate critical adaptive responses. HIF-1 was initially believed to play a crucial role in erythropoiesis by stimulating EPO production (Semenza et al. 1991). However, more recent studies have identified HIF-2 as the primary transcription factor driving EPO expression (Warnecke et al. 2004; Haase 2010). Despite this, HIF-1 remains essential for hypoxic adaptation (Alanova et al. 2024), particularly by promoting angiogenesis through VEGF upregulation, which enhances blood vessel formation and oxygen delivery (Forsythe et al. 1996). Metabolic reprogramming is another key function, ensuring anaerobic ATP production by increasing glycolytic enzymes and glucose transporters (Fukuda et al. 2007). Finally, HIF-1 supports cell survival by regulating genes that mitigate oxidative stress and sustain viability in hypoxic conditions (Ong and Hausenloy 2012). Together, these adaptations ensure effective responses to oxygen deprivation, helping cells overcome this challenge.

While HIF-1 signaling plays a critical role in cellular adaptation to hypoxia, it can be a double-edged sword. For example, in cardiomyocytes, HIF-1 activation during ischemic states promotes cell survival and initiates protective pathways that increase the ability of the heart to cope with reduced oxygen availability (Ong et al. 2014). However, in the context of cancer, the same mechanisms can be detrimental. By enhancing tumor cell survival, angiogenesis, and metabolic reprogramming, HIF-1 contributes to tumor growth, invasion, and resistance to therapy (Semenza 2003). These contrasting effects point out the tissue-specific and disease-dependent nature of HIF-1 signaling. Its adaptability underscores its importance in both beneficial physiological processes, such as cardiac protection, and pathological conditions, such as cancer progression, making it a pivotal target for therapeutic interventions across diverse medical fields, including oncology and cardiovascular medicine.

In addition to oxygen-dependent regulation, HIF-1 signaling is influenced by mechanisms operating at multiple levels, ranging from transcriptional to post-translational regulation. For example, epigenetic modifications regulate the expression of HIF-1 transcripts, while post-translational modifications such as SUMOylation and deubiquitylation modulate HIF-1 stability and activity (Koyasu et al. 2018; Catrina and Zheng 2021; Semenza 2017). This review focuses on the post-transcriptional regulation of HIF-1 by the most prevalent epitranscriptomic modification, m^6^A, with an emphasis on the bidirectional interactions between HIF-1 signaling and this RNA modification.

## N^6^-methyladenosine (m^6^A)

m^6^A is the most prevalent and well-studied epitranscriptomic modification in eukaryotic mRNAs, though it also occurs in non-coding RNAs such as long non-coding RNAs (lncRNAs), ribosomal RNAs (rRNAs), and small nuclear RNAs (snRNAs) (Zhang et al. 2021; Sendinc and Shi 2023). It is an evolutionary conserved modification found across a wide range of species, from plants to mammals, underscoring its essential role in regulating gene expression (Liang et al. 2020). Mechanistically, this modification alters RNA structure by forcing the methylamino group into an anti-conformation, destabilizing the thermodynamics of the RNA duplex (Sweaad et al. 2021). This structural change facilitates the interaction of RNA-binding proteins with their targets and subsequently influences various stages of RNA metabolism, including translation, stability, and decay (Niu et al. 2013).

**Fig. 2:**
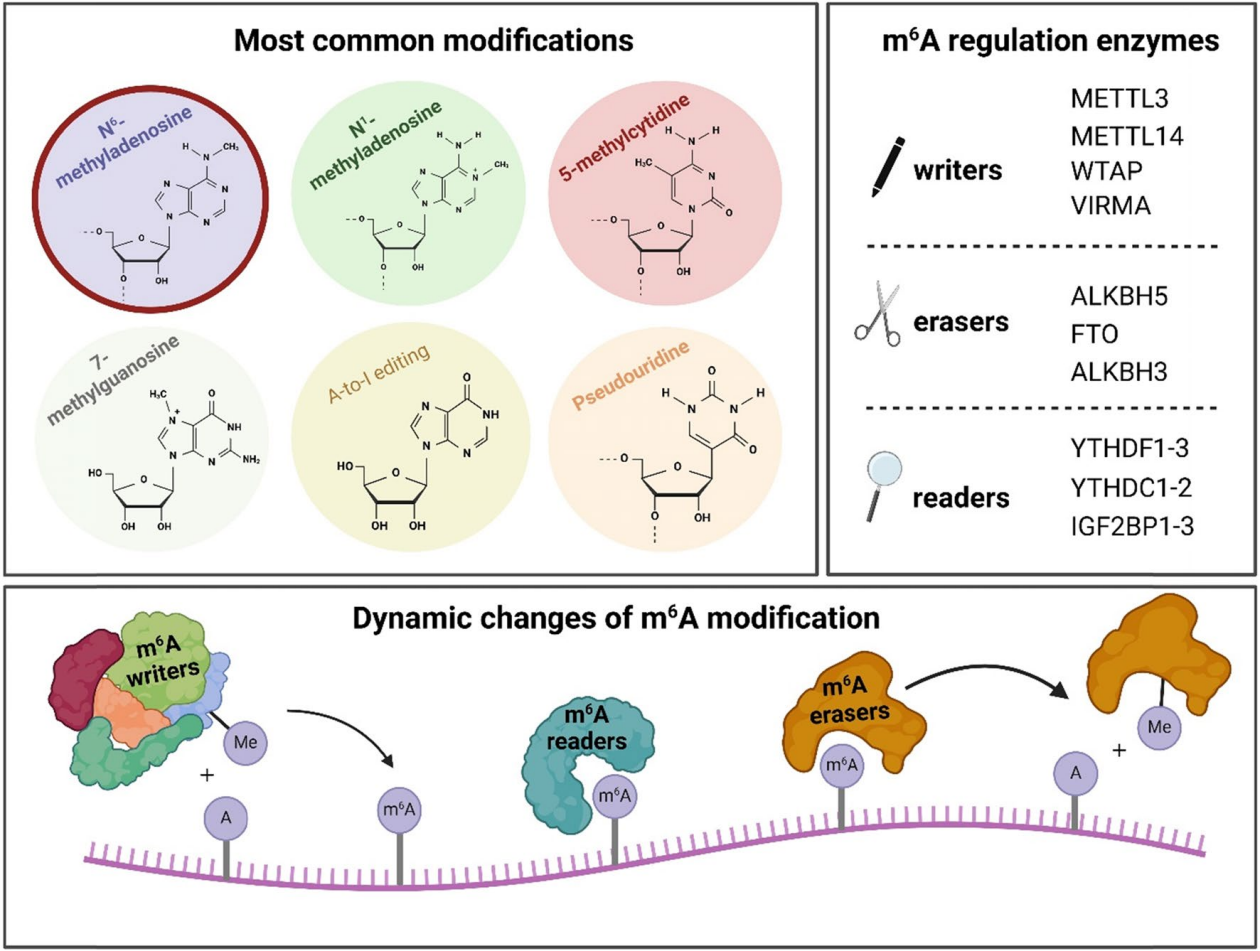
Overview of RNA modifications and m^6^A regulation. The top left panel illustrates the most common RNA modifications, including N⁶-methyladenosine, N^1^-methyladenosine, 5-methylcytidine, 7-methylguanosine, A-to-I editing, and pseudouridine. The top right panel lists key m^6^A regulatory enzymes, categorized as writers (METTL3, METTL14, WTAP, VIRMA), erasers (ALKBH5, FTO, ALKBH3), and readers (YTHDF1-3, YTHDC1-2, IGF2BP1-3). The bottom panel represents the dynamic cycle of m^6^A modification, where m^6^A writers deposit methyl groups on adenosine, readers recognize and interpret the modification, and erasers remove m^6^A to regulate RNA fate and function. ALKBH5: AlkB homolog 5; FTO: fat mass and obesity-associated; IGF2BP1-3: insulin-like growth factor 2 mRNA-binding protein 1–3; METTL3/14: methyltransferase-like 3/14; m^6^A: N^6^-methyladenosine; VIRMA: vir-like m^6^A methyltransferase associated; WTAP: Wilms’ tumor 1-associating protein; YTHDC1-2: YTH domain-containing protein 2; YTHDF1-3: YTH domain-containing family protein 1–3

The regulation of m^6^A is mediated by a group of specialized RNA-modifying enzymes (overview in Fig. 2). Writers catalyze the addition of methyl groups to RNA, creating modifications that are recognized by RNA-binding proteins known as readers. While many irreversible RNA modifications are regulated through the degradation of modified RNA, the reversible nature of m^6^A allows for its removal by specialized enzymes called erasers, enabling dynamic regulation of RNA function without requiring its decay (Benak et al. 2024a).

The primary protein complex responsible for adding the m^6^A modification to RNA consists of several key components. The most critical include methyltransferase-like 3 (METTL3), which serves as the catalytic subunit (Wang et al. 2016a, b); methyltransferase-like 14 (METTL14), which facilitates RNA binding (Wang et al. 2016a, b); and Wilms’ tumor 1-associating protein (WTAP), which aids in localizing the complex to nuclear speckles (Ping et al. 2014). Vir-like m^6^A methyltransferase associated (VIRMA, also known as KIAA1429) is another component of the methyltransferase complex, which mediates preferential methylation in specific RNA sites (Yue et al. 2018).

Several RNA-binding proteins have been identified as m^6^A readers, with the YTH domain-containing family proteins (YTHDF1-3) and YTH domain-containing proteins (YTHDC1-2) being among the most significant. These proteins play crucial roles in mRNA metabolism. While YTHDF1-3 orthologs are primarily involved in mRNA degradation (Zaccara and Jaffrey 2020; Lasman et al. 2020), both YTHDF1 and YTHDF3 have also been implicated in regulating translation (Wang et al. 2015; Shi et al. 2017). YTHDC1 is involved in mRNA splicing (Xiao et al. 2016), and YTHDC2 promotes translation (Hsu et al. 2017). IGF2BP1-3 proteins (insulin-like growth factor 2 mRNA-binding proteins 1–3) also bind to m^6^A and promote the stability and storage of their target mRNAs and therefore affect gene expression output (Huang et al. 2018).

AlkB homolog 5 (ALKBH5) and fat mass and obesity-associated protein (FTO) are the most well-known demethylases that remove the methyl group from m^6^A-modified transcripts. ALKBH5 is the primary m^6^A eraser, specifically targeting and demethylating m^6^A in mRNA and snRNA (Zheng et al. 2013; Wang et al. 2023). Although FTO is not exclusively specific to m^6^A, it predominantly acts on m^6^A in the nucleus, where it interacts with various RNA species, including mRNA, snRNA, and tRNA. In addition to m^6^A, FTO also demethylates N^6^,2ʹ-O-dimethyladenosine (m^6^Am) and N^1^-methyladenosine (m^1^A), further expanding its regulatory role in RNA metabolism (Benak et al. 2024b; Jia et al. 2011; Wei et al. 2018; Relier et al. 2021). Another eraser, AlkB homolog 3 (ALKBH3) has been reported to promote the m^6^A demethylation of mammalian tRNA (Ueda et al. 2017).

## The reciprocal relationship: HIF-1 and m^6^A

**Fig. 3:**
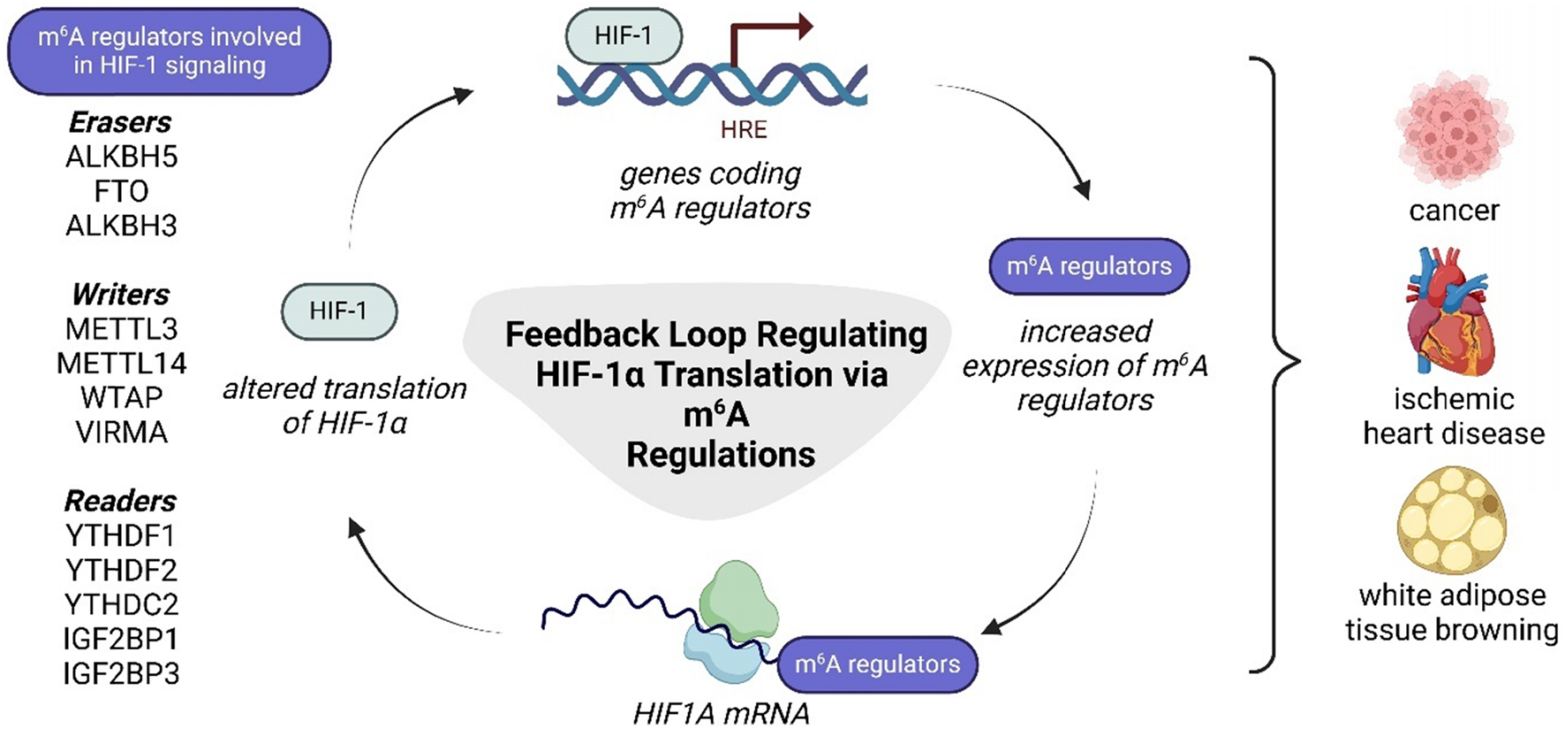
The reciprocal relationship of HIF-1 and m^6^A. This figure illustrates the interaction between m^6^A regulators and HIF-1α translation in a feedback loop. HIF-1 binds to HREs in genes encoding m^6^A writers, erasers, and readers, leading to their increased expression. These m^6^A regulators modify *HIF1A* mRNA, affecting its stability and translation, which in turn alters HIF-1α protein levels. This regulatory mechanism plays a crucial role in various physiological and pathological conditions, including cancer, ischemic heart disease, and white adipose tissue browning. ALKBH5: AlkB homolog 5; FTO: fat mass and obesity-associated; HIF-1/*HIF1A*: hypoxia-inducible factor 1; HRE: hypoxia response element; IGF2BP1/3: insulin-like growth factor 2 mRNA-binding protein 1/3; METTL3/14: methyltransferase-like 3/14; m^6^A: N^6^-methyladenosine; VIRMA: vir-like m^6^A methyltransferase associated; WTAP: Wilms’ tumor 1-associating protein; YTHDC2: YTH domain-containing protein 2; YTHDF1-2: YTH domain-containing family protein 1–2

The dynamic interaction between HIF-1 and m^6^A plays a crucial role in gene expression regulation and had been described mainly in the cancer research (overview in Fig. 3).

### HIF-1 and m^6^A erasers

One of the earliest discoveries connecting m^6^A regulators and HIF-1 involves the m^6^A demethylase ALKBH5, which was identified as a direct transcriptional target of HIF-1α already in 2011 (Thalhammer et al. 2011), two years before its role as an m^6^A demethylase was revealed (Zheng et al. 2013). In this landmark study, Thalhammer et al. (2011) demonstrated that ALKBH5 is upregulated in response to hypoxia through HIF-1α signaling. This regulation was confirmed across multiple cell lines, where ALKBH5 expression increased significantly under hypoxic conditions. Following this discovery, further research revealed that the upregulation of ALKBH5 under hypoxia contributes to cancer progression. Specifically, in breast cancer cells, ALKBH5 induces pluripotency factors, leading to the formation of a breast cancer stem cell phenotype that is essential for tumor initiation and metastasis (Zhang et al. 2016a, b). More recently, another study demonstrated that in ovarian cancer, HIF-1α-driven upregulation of ALKBH5 stimulates downstream focal adhesion kinase (FAK)/Src proto-oncogene (Src) signaling and boosts integrin subunit beta 1 (*ITGB1*) expression by disrupting the YTHDF2 protein-mediated m^6^A degradation pathway, ultimately promoting lymph node metastasis and lymphangiogenesis (Sun et al. 2023).

Furthermore, recent studies indicated that ALKBH5-mediated m^6^A demethylation also plays a critical role in regulating *HIF1A* transcription, suggesting a reciprocal relationship between m^6^A and HIF-1. ALKBH5 was found to regulate m^6^A modifications on circular RNA circCCDC134 in cervical cancer. ALKBH5-mediated demethylation of circCCDC134 enhanced its stability, enabling it to recruit the transcription factor p65 (a subunit of NF-κB) and to act as a miR-503-5p sponge. These processes promoted the transcription of *HIF1A*, contributing to cancer progression and metastasis (Liang et al. 2022).

In addition to ALKBH5, also FTO plays a significant role in the regulation of HIF-1. FTO is upregulated in various cancers and contributes to tumor progression by influencing HIF-1 activity. One key study demonstrated that FTO demethylates and stabilizes the long intergenic non-coding RNA for kinase activation (*LINK-A*) in esophageal squamous cell carcinoma. *LINK-A*, in turn, disrupts the interaction between minichromosome maintenance complex component 3 (MCM3) and HIF-1α, abrogating MCM3-mediated HIF-1α transcriptional repression and promoting glycolysis and chemoresistance (Nan et al. 2023).

Beyond the direct regulation of HIF-1 by FTO, these two proteins also act synergistically. This was demonstrated with the hypoxia-responsive gene aldolase A (*ALDOA*), whose transcription is increased by HIF-1α under low oxygen conditions. At the same time, FTO demethylates *ALDOA* transcripts, stabilizing them and promoting their persistence, ultimately leading to the metabolic adaptation of cancer cells to hypoxia, enhancing glycolysis and hepatocellular carcinoma (HCC) progression (Niu et al. 2021).

These findings underscore the critical roles of m^6^A demethylases, ALKBH5 and FTO, in regulating HIF-1α activity. Both enzymes enhance the stability of key transcripts, like circCCDC134 and *LINK-A*, driving cancer progression. Moreover, these erasers can act synergistically with HIF-1α, as demonstrated with *ALDOA*, promoting hypoxia adaptation and tumor growth. These insights highlight the potential of targeting m^6^A demethylation in cancers dependent on HIF-1 signaling.

Moreover, influence of FTO on HIF-1α extends beyond cancer, playing a pivotal role in cardiovascular disorders, including ischemic heart disease and myocardial infarction (MI), both of which are associated with hypoxia. In MI, FTO expression was significantly downregulated in affected tissues, suggesting its potential protective role in cardiac injury (Shi et al. 2021; Wang et al. 2024). Dysregulation of the m^6^A machinery, including FTO, has been linked to key pathways such as PI3K-Akt and HIF-1, highlighting capacity of FTO to modulate cardiac function and hypoxic responses in ischemic conditions (Shi et al. 2021). In neonatal cardiac fibroblasts exposed to hypoxia (1% O₂ for 24 h), a decrease in FTO levels was associated with an increase in HIF-1α levels. Further experiments revealed a reciprocal regulatory mechanism wherein HIF-1α bound to HREs in the *Fto* promoter, suppressing its expression. In contrast, inhibition or knockdown of HIF-1α restored FTO expression, underlining their dynamic interplay (Wang et al. 2024). These findings are particularly intriguing, as HIF-1 is traditionally associated with transcriptional activation rather than suppression. Interestingly, in fasting induced cardioprotection, FTO levels were markedly increased, while HIF-1α levels were reduced at both transcript and protein levels in the heart (Benak et al. 2024c, d). Despite this downregulation, the m^6^A/m (m^6^A + m^6^Am) methylation levels on *Hif1a* mRNA remained stable, showing only a non-significant trend toward increased methylation (Benak et al. 2024c). These findings emphasize the intricate relationship between HIF-1 and FTO in cardiovascular contexts.

Another study showed that FTO deficiency in metabolic contexts, such as white adipose tissue browning, increases m^6^A methylation on *Hif1a* mRNA. This methylation, recognized by YTHDC2, promotes *Hif1a* translation and enhances HIF-1α protein levels, thereby activating thermogenic genes. This, in turn, promotes the white-to-beige fat transition and thermogenesis, leading to increased energy expenditure and protection against diet-induced obesity (Wu et al. 2021).

The association between HIF-1 and the third known demethylase, ALKBH3, remains poorly understood. However, one study reported that HIF-1α transcriptionally activates the expression of the lncRNA *ALKBH3-AS1*, which stabilizes ALKBH3 mRNA and promotes HCC cell proliferation and invasion (Lu et al. 2022).

Currently, all three known m^6^A erasers have been implicated in the regulation of HIF-1 signaling, drawing attention to their significant roles in cancer progression, energy homeostasis, and cardiovascular resistance to ischemia.

### HIF-1 and m^6^A writers

Since the association of m^6^A erasers on HIF-1 signaling was established, it was only a matter of time to reveal the involvement of m^6^A writers as well. Similarly to erasers, these connections were mostly studied in cancer contexts.

METTL3, the most extensively studied m^6^A writer, has been shown to promote HCC progression by mediating m^6^A modifications on *HIF1A* mRNA, resulting in reprogramming of cellular metabolism, as well as enhanced proliferation, invasion and metastasis of liver cancer (Yang et al. 2021a; Zhang et al. 2024). In colorectal cancer, which is characterized by elevated METTL3 and HIF-1α levels, *METTL3* knockdown reduced the m^6^A modification of *HIF1A* and lowered its translation efficiency, leading to the suppression of the Warburg effect. Moreover, this group also reported that HIF-1α binds to two main HREs in the promoter of *METTL3*, inducing its expression under hypoxia (Yang et al. 2021b). Similarly, in arecoline-induced oral squamous cell carcinoma, elevated METTL3 expression is stimulated by HIF-1α, forming a positive autoregulatory loop with MYC proto-oncogene that influences both carcinogenesis and cisplatin resistance (Wang et al. 2022). Beyond these direct interactions, METTL3 also promotes the m^6^A-dependent miR-21-5p maturation, which targets hypoxia-inducible factor 1 subunit alpha inhibitor (*HIF1AN*; gene name for FIH-1), a negative regulator of HIF-1α. This results in the activation of the HIF-1/VEGF signaling axis, promoting choriocarcinoma progression (Ye et al. 2022). A recent study revealed the role of the HIF-1α/METTL3/YTHDF2 axis in inhibiting the NF-κB/CCL3 pathway, contributing to benzene-induced hematotoxicity (Cong et al. 2024). This suggests that METTL3’s involvement in HIF-1 signaling extends beyond cancer, influencing immune and hematopoietic responses under toxic conditions.

Other subunits of the m^6^A methyltransferase complex are associated with HIF-1 signaling as well. METTL14-induced ferroptosis, mediated through m^6^A-YTHDF2-dependent degradation of solute carrier family 7 member 11 (*SLC7A11*), is dramatically abolished in hypoxic environment in a HIF-1α-dependent manner, contributing to HCC progression (Fan et al. 2021). *WTAP*, a key subunit of the m^6^A methyltransferase complex, is upregulated by HIF-1α in ovarian cancer, where it promotes the Warburg effect by stabilizing the mRNA of the glycolytic enzyme hexokinase 2 (HK2) through miR-200 in an m^6^A-dependent manner, thereby accelerating tumor progression under hypoxic conditions (Lyu et al. 2022). In acute myeloid leukemia (AML), WTAP is similarly up-regulated by HIF-1α and stabilizes lysine demethylase 4B (*KDM4B*) mRNA via m^6^A modification, driving cancer cell proliferation and survival (Shao et al. 2023). VIRMA is up-regulated in colon adenocarcinoma, and its silencing in colonic adenocarcinoma cell lines has been shown to inhibit tumor growth by blocking the HIF-1 signaling pathway. However, reactivation of the HIF-1 signaling pathway can counteract the antitumor effects of *VIRMA* silencing (Ouyang et al. 2024). Similarly, VIRMA is highly expressed in pancreatic ductal adenocarcinoma and is associated with poor prognosis. Mechanistically, VIRMA is promoting signaling through the STRA6/STAT3 axis, leading to elevated HIF-1α levels, which in turn enhances glycolysis and drives tumor progression (Yang et al. 2024).

Currently, multiple m^6^A writers, including METTL3, METTL14, WTAP, and VIRMA, have been shown to play critical roles in HIF-1 signaling, primarily in cancer progression. Their involvement influences metabolic reprogramming, tumor proliferation, and adaptation to hypoxia, underlining the significance of m^6^A methylation in regulating HIF-1 signaling and its potential as a therapeutic target in cancer.

### HIF-1 and m^6^A readers

Compared to m^6^A writers and erasers, the relationship between m^6^A readers and HIF-1 signaling remains less explored. However, as mediators of RNA modification functions, m^6^A readers play a crucial role in shaping cellular responses to hypoxia and metabolic stress.

Among key m^6^A readers, YTHDF1 is directly regulated under hypoxic conditions. HIF-1α induces YTHDF1 expression, which in turn drives hypoxia-induced autophagy and malignancy of HCC by promoting translation of autophagy-related genes *ATG2A* and *ATG14* in a m^6^A-dependent manner (Li et al. 2021). However, the regulation of YTHDF1 in hypoxia appears complex. In contrast to its upregulation in HCC, *YTHDF1* levels are reduced in both the kidneys and liver of highland cattle compared to lowland cattle, suggesting context-dependent negative regulation in hypoxia (Shi et al. 2019). Another key reader, YTHDF2, cooperates with polybromo 1 (PBRM1) to regulate HIF-1α protein translation (Shmakova et al. 2021), which is also promoted by reader YTHDC2 (Wu et al. 2021).

Beyond the YTH domain family, HIF-1α-dependent upregulation of m^6^A reader IGF2BP1 has been also reported. The study revealed that this axis facilitates peripheral nerve injury recovery by enhancing *SLC7A11* mRNA stabilization (An et al. 2023). Reader IGF2BP3 is highly expressed in stomach cancer tissues and hypoxia-treated stomach cancer cells alongside HIF-1α. Mechanistically, IGF2BP3 directly binds to an m^6^A site within the *HIF1A* mRNA coding region, positively regulating its expression. Knockdown of *IGF2BP3* inhibits hypoxia-induced cell migration and angiogenesis by modulating HIF-1α in stomach cancer (Jiang et al. 2021). Additionally, IGF2BP3 stabilizes *HIF1A* mRNA promoting its expression during hepatocyte reprogramming in acute-on-chronic liver failure (Cheng et al. 2023).

These findings accentuate the dynamic and context-dependent interaction between HIF-1 signaling and m^6^A readers, highlighting their critical role in fine-tuning hypoxic adaptation and disease progression.

Collectively, current data bring attention to the intricate crosstalk between HIF-1 signaling and m^6^A erasers, writers, and readers. A summary of these bidirectional regulatory pathways is provided in Table 1.

**Table 1:** Summary of bidirectional regulatory pathways between HIF-1 and m^6^A RNA modification regulators

m^6^A regulator	Cell/tissue type	Regulation	Ref
HIF-1 → regulation of m^6^A pathway			
ALKBH5	MCF7, U2OS, and IMR32 cells	hypoxia → HIF-1 ↑ → *ALKHBH5 ↑*	Thalhammer et al. (2011)
FTO	Neonatal cardiac fibroblasts	hypoxia → HIF-1 ↑ → *Fto* ↓	Wang et al. (2024)
METTL3	CRC cell lines	hypoxia → HIF-1 ↑ → *METTL3* ↑	Yang et al. (2021b)
	Arecoline-induced oral squamous cell carcinoma	HIF-1 ↑ → METTL3 ↑ → tumorigenesis and cisplatin resistance	Wang et al. (2022)
METTL14	Hepatocellular carcinoma	hypoxia → HIF-1 ↑ → METTL14 ↓ → abrogated ferroptosis and cancer progression	Fan et al. (2021)
WTAP	Ovarian cancer	HIF-1 ↑ → WTAP ↑ → Warburg effect and cancer progression	Lyu et al. (2022)
	Acute myeloid leukemia	HIF-1 ↑ → WTAP ↑ → cancer progression	Shao et al. (2023)
YTHDF1	Hepatocellular carcinoma	HIF-1 ↑ → YTHDF1 ↑ → autophagy and cancer progression	Li et al. (2021)
IGF2BP1	Dorsal root ganglion neurons	hypoxia → HIF-1 ↑ → *IGF2BP1 ↑* → peripheral nerve injury recovery	An et al. (2023)
regulation of m^6^A pathway → HIF-1			
FTO	Mouse adipose tissue	*FTO* deletion → m^6^A levels on *Hif1a ↑ → *YTHDC2 binding → HIF-1α ↑ → adipocyte browning	Wu et al. (2021)
METTL3	Hepatocellular carcinoma	METTL3 ↑ → m^6^A levels on *HIF1A ↑* → cancer progression	Yang et al. (2021a), Zhang et al. (2024)
	Colorectal cancer	*METTL3* knockdown → m^6^A levels on *HIF1A* ↓ → HIF-1α ↓ → Warburg effect ↑	Yang et al. (2021b)
VIRMA	Colon adenocarcinoma	VIRMA ↑ → *VIRMA* silencing → HIF-1 signaling ↓ → tumor growth inhibition	Ouyang et al. (2024)
	Pancreatic ductal adenocarcinoma	VIRMA ↑ → HIF-1α ↑ → cancer progression	Yang et al. (2024)
YTHDF2	HeLa and H1299 cells	MG132 treatment + *YTHDF2* silencing → HIF-1α ↓	Shmakova et al. (2021)
YTHDC2	Mouse adipose tissue	*FTO* deletion → m^6^A levels on *Hif1a ↑ → *YTHDC2 binding → HIF-1α ↑ → adipocyte browning	Wu et al. (2021)
IGF2BP3	Stomach cancer	IGF2BP3 ↑→ HIF-1α ↑ → hypoxia-induced cell migration and angiogenesis	Jiang et al. (2021)
	Hep3B and HepG2 cells	*IGF2BP3* silencing → HIF-1α ↓	Cheng et al. (2023)

ALKBH5: AlkB homolog 5; CRC: colorectal cancer; FTO: fat mass and obesity-associated; HIF-1/*HIF1A*: hypoxia-inducible factor 1; IGF2BP1/3: insulin-like growth factor 2 mRNA-binding protein; METTL3/14: methyltransferase-like 3/14; m^6^A: N^6^-methyladenosine; VIRMA: protein virilizer homolog; WTAP: Wilms’ tumor 1-associating protein; YTHDC2: YTH domain-containing protein 2; YTHDF1-2: YTH domain-containing family protein 1–2

## HIF-1 and other RNA modifications

Besides m^6^A, the reciprocal relationship between RNA modifications and HIF-1 remains relatively unexplored. Notably, two negative regulators of HIF-1α—the natural antisense transcript *HIF1A-AS2* and ubiquitin ligase scaffold *LIMD1*—have been identified as targets of adenosine-to-inosine (A-to-I) RNA editing, mediated by adenosine deaminase acting on RNA 1 (ADAR1). This ADAR1-dependent modification facilitates the robust and timely accumulation of HIF-1α upon oxygen depletion, thereby reinforcing downstream angiogenesis (Ma et al. 2019). In renal cell carcinoma cells, it has been shown that HIF-1 accumulation leads to downregulation of pseudouridine synthase 10 (PUS10) (Luo et al. 2023), an enzyme responsible for converting uridine to pseudouridine (Ψ) in tRNA (Gurha and Gupta 2008). More recently, a study on colorectal cancer cells reported that HIF-1 binds HRE in the promoter region of methyltransferase *METTL1*, ultimately leading to inhibition of its transcription and subsequent decrease in the levels of N^7^-methylguanosine (m^7^G) in tRNA (Mi et al. 2024).

The demethylase ALKBH1, known for its role in RNA modification such as N^1^-methyladenosine (m^1^A), 5-methylcytidine (m^5^C), or N^3^-methylcytidine (m^3^C), has also been implicated in HIF-1 signaling (Zhong et al. 2024; Wu et al. 2019; Guo et al. 2020; Liu et al. 2022). However, ALKBH1 can demethylate not only RNA but also DNA modifications, including N^6^-methyldeoxyadenosine (6mA) (Zhong et al. 2024). Notably, ALKBH1-mediated 6mA DNA demethylation within the promoter of HIF-1 target gene *MIAT* (lncRNA myocardial infarction-associated transcript) facilitates HIF-1 binding and enhances its transcriptional activation (Wu et al. 2019). Additionally, direct ALKBH1-mediated regulation of 6mA in the *HIF1A* gene has been observed (Guo et al. 2020; Liu et al. 2022). These findings suggest that ALKBH1 primarily regulates HIF-1 signaling through epigenetic (DNA) modifications rather than an epitranscriptomic (RNA) mechanism. However, given its broad activity across both RNA and DNA substrates, the potential for RNA modification-mediated effects on HIF-1 remains an open question.

A similar issue arises with ten-eleven translocation (TET) proteins, which function as erasers of m^5^C in RNA as well as 5-methyl-2′-deoxycytidine (5mdC) in DNA. All TET proteins (TET1-3) have been implicated in hypoxia-related responses (Cheng et al. 2018; Zhang et al. 2022; Cao et al. 2020). However, while their expression is regulated in a HIF-1-dependent manner (Cao et al. 2020; Ma et al. 2019; Hains et al. 2022), the reciprocal TET-HIF axis is mediated by DNA hydroxymethylation regulation rather than epitranscriptomic changes (Cheng et al. 2018; Cao et al. 2020; Ma et al. 2019). Thus, further research is needed to determine whether TET enzymes participate in hypoxia-driven RNA epitranscriptomic modifications, akin to their role in DNA demethylation.

While m^6^A remains the most studied RNA modification in the context of HIF-1 regulation, emerging evidence suggests that other epitranscriptomic marks, including A-to-I editing, Ψ, and m^7^G, may also contribute to hypoxia responses. At the same time, enzymes such as ALKBH1 and TET proteins, though primarily studied in DNA modification, may have underexplored roles in RNA demethylation under hypoxia. The reciprocal relationship between HIF-1 and these RNA modifications remains unresolved, highlighting the need for further research to uncover the full extent of epitranscriptomic regulation in hypoxic signaling.

## Clinical implications

Hypoxia is a defining characteristic of the tumor microenvironment, arising as rapidly proliferating cancer cells outpace their blood supply. To survive and proliferate under these conditions, cancer cells rely on HIF-1α, a master regulator of the adaptive response to low oxygen. HIF-1α drives the transcription of genes involved in angiogenesis, glycolysis, and cell survival (Semenza 2003). Concurrently, epitranscriptomic modifications, particularly m^6^A methylation, modulate the HIF-1 signaling pathway, contributing to the malignant progression of tumors. Beyond cancer, epitranscriptomic regulation and the HIF-1 pathway are implicated in hypoxia-driven cardioprotection, such as the adaptive response to ischemia–reperfusion injury. As such, targeting these regulatory systems could present innovative therapeutic strategies to both inhibit cancer progression and address ischemic heart disease—two major global health challenges.

HIF-1α has been a focal point for drug development. Inhibitors such as topotecan (Parmakhtiar et al. 2019) or PX-478 (Jacoby et al. 2010) and activators of PHDs like KRH102053 (Choi et al. 2008) or KRH102140 (Nepal et al. 2011) have shown efficacy in tumor cells. Despite these advances, these compounds often suffer from off-target effects, toxicity to normal cells, low selectivity, and the formation of toxic metabolites. These limitations stress the need for innovative strategies to enhance the specificity and safety of such compounds.

Recent studies have also demonstrated the potential of small molecule inhibitors targeting m^6^A regulators *in vitro* and in animal studies (An and Duan 2022). Recently, STC-15, an inhibitor of the m^6^A writer METTL3, has become the first RNA-modifying enzyme inhibitor to enter clinical trials (NCT05584111) for cancer treatment (Medicine NLo, 2022). However, inhibitors of other m^6^A regulators have not yet reached this stage. Several hurdles remain before these inhibitors can be effectively translated into clinical practice. Epitranscriptomic regulators influence a broad array of cellular processes beyond their roles in cancer and ischemia, creating a significant risk of off-target effects. The widespread involvement of these enzymes in RNA metabolism makes selective targeting of disease-relevant pathways a critical but unmet need.

The substrate selectivity and dynamics of m^6^A regulators are still not well understood, raising questions whether their activity depends on sequence specificity (Li et al. 2019), subcellular localization (Relier et al. 2022), or regulation by specific proteins (Nabeel-Shah et al. 2024). Recent work by He et al. (He et al. 2023) elucidated the mechanisms by which m^6^A is selectively deposited and suppressed on mRNA, emphasizing the role of exon junction complexes (EJCs) in this regulation. Their findings demonstrate that EJCs act as suppressors of m^6^A methylation, particularly near exon-exon junctions, which limit methylation to certain regions of the mRNA transcript. This regulation ensures that only specific transcripts are targeted for m^6^A modifications, a process that is critical for maintaining stability and proper expression of mRNAs. Another current study demonstrated that the interaction between FTO and its target transcripts can be modulated by the telomeric zinc finger protein ZBTB48, uncovering a previously unrecognized mechanism that regulates FTO effects on RNA expression (Nabeel-Shah et al. 2024).

Moving forward, comprehensive basic research is paramount to address these challenges. Investigating the interaction between epitranscriptomics and HIF-1 signaling under pathological conditions will enable the identification of precise therapeutic windows. Furthermore, integrating cutting-edge technologies such as single-cell transcriptomics and CRISPR-based screens could accelerate the discovery of disease-specific regulatory networks. Such efforts will be instrumental in transforming promising preclinical findings into viable clinical interventions, offering new treatment for patients with cancer and ischemic heart disease.

## Conclusions

The growing body of research on the interplay between RNA modifications, especially m^6^A, and HIF-1 signaling underscores the complexity of cellular responses to oxygen deprivation. While HIF-1 has long been recognized as a central regulator in hypoxia-driven processes, the recent discovery of its interaction with epitranscriptomic modulators offers new insights into the fine-tuning of gene expression. A particularly intriguing aspect is that many epitranscriptomic regulators not only influence HIF-1 signaling but may also be direct targets of HIF-1 itself. The bidirectional regulation between m^6^A modifications and HIF signaling not only enhances our understanding of cellular hypoxic responses but also points to potential therapeutic strategies for diseases such as cancer, ischemic heart disease, and other pathologies linked to dysregulated oxygen homeostasis.

## Data Availability

No datasets were generated or analysed during the current study.

## Abbreviations

5mdC: 5-methyl-2′-deoxycytidine
6mA: N^6^-methyldeoxyadenosine
ADAR1: Adenosine deaminase acting on RNA 1
ALDOA: Aldolase A
ALKBH1/3/5: AlkB homolog 1/3/5
AML: Acute myeloid leukemia
ARNT: Aryl hydrocarbon receptor nuclear translocator
ATG: Autophagy-related genes
CCDC: Coiled-coil domain-containing
CCL: Chemokine (C–C motif) ligand
EJC: Exon junction complex
EPO: Erythropoietin
FIH: Factor inhibiting HIF
FIH-1: Factor inhibiting HIF-1
FTO: Fat mass and obesity-associated protein
HCC: Hepatocellular carcinoma
HIF-1: Hypoxia-inducible factor 1
HK2: Hexokinase 2
HRE: Hypoxia-responsive element
IGF2BP: Insulin-like growth factor 2 mRNA-binding protein
KDM: Lysine demethylase
lncRNA: Long non-coding RNA
MCM3: Minichromosome maintenance complex component 3
METTL1/3/14: Methyltransferase-like 1/3/14
MI: Myocardial infarction
m^1^A: N^1^-methyladenosine
m^6^A: N^6^-methyladenosine
m^6^Am: N^6^, 2’-O-dimethyladenosine
m^3^C: N^3^-methylcytidine
m^5^C: 5-methylcytidine
m^7^G: N^7^-methylguanosine
*MIAT*: Myocardial infarction-associated transcript
NF-κB: Nuclear factor kappa B
PI3K-Akt: Phosphoinositide 3-kinase–protein kinase B pathway
PHD: Prolyl hydroxylase domain
PBRM1: Protein polybromo 1
pVHL: Von Hippel-Lindau protein
SLC: Solute carrier family
snRNA: Small nuclear RNA
STRA6: Signaling receptor and transporter of retinol STRA6
TET: Ten-eleven translocation proteins
VEGF: Vascular endothelial growth factor
VIRMA: Vir-like m^6^A methyltransferase associated
WTAP: Wilms’ tumor 1-associating protein
YTHDC1-2: YTH domain-containing protein 1–2
YTHDF1-3: YTH domain-containing family protein 1–3
ZBTB48: Zinc finger and BTB domain-containing protein 48
Ψ: Pseudouridine
